# Quantum image distillation

**DOI:** 10.1126/sciadv.aax0307

**Published:** 2019-10-18

**Authors:** Hugo Defienne, Matthew Reichert, Jason W. Fleischer, Daniele Faccio

**Affiliations:** 1 School of Physics and Astronomy, University of Glasgow, Glasgow G12 8QQ, UK.; 2 Department of Electrical Engineering, Princeton University, Princeton, NJ 08544, USA.

## Abstract

Imaging with quantum states of light promises advantages over classical approaches in terms of resolution, signal-to-noise ratio, and sensitivity. However, quantum detectors are particularly sensitive sources of classical noise that can reduce or cancel any quantum advantage in the final result. Without operating in the single-photon counting regime, we experimentally demonstrate distillation of a quantum image from measured data composed of a superposition of both quantum and classical light. We measure the image of an object formed under quantum illumination (correlated photons) that is mixed with another image produced by classical light (uncorrelated photons) with the same spectrum and polarization, and we demonstrate near-perfect separation of the two superimposed images by intensity correlation measurements. This work provides a method to mix and distinguish information carried by quantum and classical light, which may be useful for quantum imaging, communications, and security.

## Teaser

Images composed of both quantum and classical light can be distilled so as to separate the quantum from the classical information.

## INTRODUCTION

Quantum imaging exploits photon correlations to overcome fundamental limits of classical imaging. Spatial correlations between pairs of photons are particularly attractive owing to their natural high-dimensional structure (*1*–*3*) and the simplicity of photon-pair generation from spontaneous parametric down-conversion (SPDC) (*4*). Demonstrations using spatially entangled photon pairs range from ghost imaging (*5*) to subshot-noise imaging (*6*, *7*) and enhanced-resolution imaging (*8*). In recent years, important progress has been made in quantum light detection to develop applications from these proof-of-principle experiments. In that regard, multipixel single-photon sensitive cameras, such as thresholded electron multiplied charge coupled device (EMCCD) (*9*) and single-photon avalanche photodiode (SPAD) cameras (*10*), have demonstrated great potential to perform high-dimensional coincidence measurements for entanglement characterization (*11*–*13*), sub-Rayleigh imaging (*14*), and super-resolution microscopy (*15*, *16*). However, all these quantum detectors operate in the single-photon counting regime (i.e., photons detected one by one), making them extremely vulnerable to sources of classical noise (e.g., background illumination, spurious reflection, etc.). For example, an excess of spurious photons detected in a SPAD-based quantum imaging system (*17*) is likely to saturate the sensor and severely hinder its use. To date, there is still no obvious means of distinguishing a quantum image from classical noise or from a superimposed classical image. Moreover, this problem extends beyond imaging and is tightly related to quantum-classical information discrimination in communications and cryptography (*18*).

Here, we report an experimental technique that allows the distillation of a quantum image from a camera measurement that contains both a quantum and a classical image. No prior information of the images themselves is required other than the statistics of the illuminating sources (i.e., the quantum image is encoded in correlated photon-pair events). An object illuminated by spatially entangled photon pairs forms an image that is mixed with that of another object illuminated by classical coherent light. Both images are indistinguishable in terms of spectrum and polarization, so that conventional intensity measurements cannot discern between them. However, intensity correlation measurements are sensitive to photon statistics. While photons emitted by the classical coherent source are uncorrelated (*19*), pairs of photons in the SPDC illumination are correlated in position (*20*, *21*). We exploit these spatial intensity correlations to extract an image of the object illuminated by photon pairs from a mixed quantum-classical image and thus reconstruct the classical image by subtraction. We lastly investigate the impact of classical light on the signal-to-noise ratio (SNR) and show that quantum information can be retrieved even when the classical illumination is 10 times higher than the quantum illumination.

**Fig. 1:**
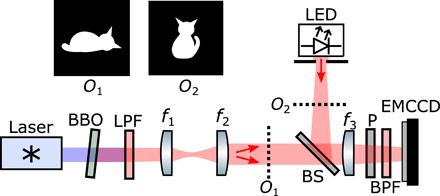
Experimental apparatus. Light emitted by a diode laser (λ*_p_* = 405 nm) illuminates β-barium borate (BBO) crystal with a thickness of 0.5 mm to produce spatially entangled pairs of photons by type I SPDC. Long-pass filters (LPF) positioned after the crystal remove pump photons. Lenses *f*_1_ = 35 mm and *f*_2_ = 75 mm image the crystal surface onto an object *O*_1_ (dead cat). Simultaneously, an object *O*_2_ (alive cat) is illuminated by a spatially filtered light-emitting diode (LED). Images of both objects are superimposed onto an EMCCD camera using a single-lens imaging configuration (*f*_3_ = 50 mm) and an unbalanced beam splitter (BS; 92% transmission). Band-pass filters (BPF) at 810 ± 5 nm and a polarizer (P) in front of the camera select near-degenerate photons. The single and double red arrows indicate respectively classical and photon-pair illuminations.

Figure 1 shows the experimental setup. A collimated laser beam (405 nm) interacts with a tilted nonlinear crystal of β-barium borate to produce pairs of infrared photons by type I SPDC. The down-converted field at the output of the crystal is imaged onto an object *O*_1_ (“dead cat”) using a two-lens imaging system *f*_1_ − *f*_2_. Simultaneously, a spatially filtered light-emitting diode (LED) illuminates a second object *O*_2_ (“alive cat”). A single-lens imaging system (*f*_3_) and an unbalanced beam splitter (92% transmission) image both objects onto an EMCCD camera. Narrowband-pass filters and polarizers ensure that all photons falling on the camera sensor have the same wavelength (810 ± 5 nm) and polarization.

## RESULTS

**Fig. 2:**
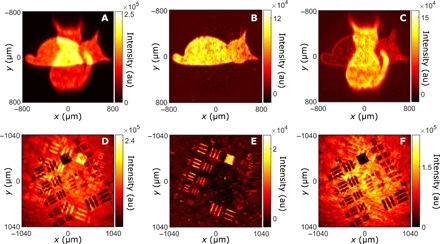
Separation of mixed quantum-classical images. The direct-intensity image (**A**) acquired by accumulating photons on the camera sensor shows a superposition of both objects *O*_1_ (quantum) and *O*_2_ (classical), representing a dead cat and an alive cat, respectively. Intensity correlation function Γ(**r**, **r**) (**B**) measured with the camera shows the image of *O*_1_. An image of *O*_2_ (**C**) is obtained by subtracting the reconstructed image of *O*_1_ from the mixed image. The residual image of *O*_1_ observed in the background is due to single photons created by absorption of one photon of a pair propagating through the dead cat mask. A similar experiment is performed using positive (*O*_1_) and negative (*O*_2_) resolution charts, as shown by its corresponding (**D**) direct-intensity image, (**E**) Γ(**r**, **r**), and (**F**) reconstructed classical image. Both experiments are performed by acquiring *N* ∼ 10^7^ frames using an exposure time of τ = 6 ms. a.u, arbitrary units.

Figure 2A shows an intensity image acquired by photon accumulation on the camera under simultaneous illumination from both sources. Objects *O*_1_ and *O*_2_ (i.e., both the dead and alive cats) are superimposed. Figure 2B shows an image of Γ(**r**, **r**), where Γ is the intensity correlation function and **r** is a camera pixel position. As detailed in Methods, Γ is retrieved using the full dynamic range of the camera (i.e., no photon counting), which prevents the sensor from saturating because of multiple photon detections. Only the object *O*_1_ that is illuminated by down-converted light (i.e., the dead cat) is apparent. Since photons emitted by the classical source are uncorrelated, the only non-null contribution to Γ is due to entangled photon pairs produced by SPDC. When pairs of photons correlated in position illuminate homogeneously, an object *O*_1_ (*22*), Γ(**r**, **r**), is proportional to its shape.

$$\Gamma (r,r)∼{|O_{1}(r)|}^{4}$$ (1)

Not only does this approach allow near-perfect reconstruction of the quantum image, but it also enables to retrieve the classical image (i.e., alive cat) by subtracting the quantum image (Fig. 2B) from the mixed image (Fig. 2A), as shown in Fig. 2C. The same experiment performed with more complex objects (i.e., resolution charts in Fig. 2D) continues to show a very good extraction of the quantum image (Fig. 2E). However, we observe the presence of residual intensities in the retrieved classical images (Fig. 2, C and F) that are located near the edges and in the head of the dead cat mask. This effect is due to single photons created by absorption of one photon of a pair when propagating through the objects (*23*).

**Fig. 3:**
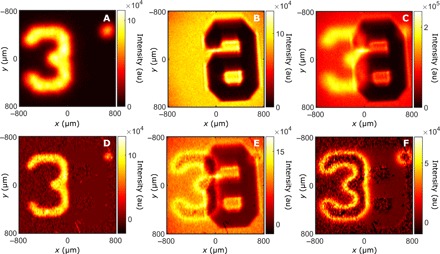
Characterization of residual single-photon intensity. Direct-intensity image (**A**) acquired with the LED turned off shows object *O*_3_ (the number “3”). The image is deliberately slightly defocused by positioning it out of the focal plane of the imaging system. Direct-intensity image (**B**) acquired with the SPDC turned off shows the ground-truth image of *O*_4_ (the number “6”). Direct-intensity image (**C**) acquired with both sources on shows a superimposition of both objects. The intensity correlation function Γ(**r**, **r**) (**D**) reveals the number “3”; image subtraction between this and the mixed image reveals the classical image (**E**) number “6.” In this case, the residual intensity created by absorption of one photon of a pair is concentrated near the edge of the number “3.” The residual single-photon intensity (**F**) is isolated by subtracting the reconstructed classical (E) from its ground truth (B). Experiments are performed by acquiring *N* = 6 × 10^6^ frames using an exposure time of τ = 6 ms.

These residual single-photon intensities are further investigated by performing a similar experiment using another object *O*_3_ (a number “3”) that is purposely positioned slightly out of the focal plane of the imaging system. A ground-truth intensity image (Fig. 3A, acquired with the LED turned off) shows the slightly defocused image of *O*_3_, well recognizable by its blurred edges. After turning on the LED, the mixed-intensity image (Fig. 3C) shows a superposition of the number “3” with a number “6” (object *O*_4_). While the number “3” is near-perfectly reconstructed by measuring Γ(**r**, **r**) (Fig. 3D), we again observe residual intensities in the classical image obtained by image subtraction (Fig. 3E). Subtracting this image from the ground truth of *O*_4_ (Fig. 3B, acquired with the photon-pair source turned off) allows us to isolate the residual intensity pattern (Fig. 3F). First, we observe that the residual edges of number “3” are thicker than edges of the dead cat in Fig. 2C. Pairs of photons out of the focal plane have a larger correlation width (*23*) and therefore a higher probability that one of them gets blocked by the object. Then, the absence of residual intensity inside the “3” is due to the near-perfect transparency at 810 nm of the printed glass (Thorlabs resolution target). These observations confirm that the residual intensity is not a detection artifact but corresponds to the physical absorption of one photon of a pair when interacting with the object. Because spatial correlations are absent from both single-photon beams and photons emitted by classical light, our intensity-correlation–based approach cannot distinguish between them, preventing us from achieving perfect reconstruction of *O*_4_ from the mixed image.

**Fig. 4:**
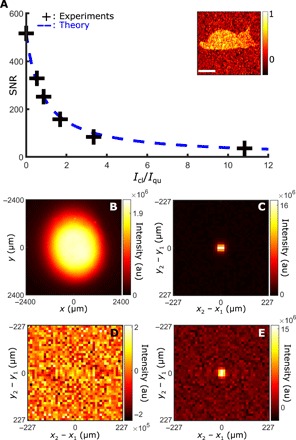
SNR in quantum-distilled images. (**A**) SNRs are represented as a function of average intensity ratio between classical and quantum light *I*_cl_/*I*_qu_ (black crosses) together with a theoretical model (blue dashed line). In this experiment, both sources homogeneously illuminate the camera sensor (**B**) and SNRs are measured by dividing the peak intensity by the SD of the noise in the minus-coordinate projections of Γ. (B) (**C**), and (**D**) show minus-coordinate projections acquired for intensity ratios of 0, +∞, and 11, respectively. All experiments are performed by acquiring *N* = 251,600 images with an exposure time of τ = 6 ms. With these settings, intensity of the quantum source averaged over camera pixels is equal to *I*_qu_ = 939 gl. Inset, normalized quantum image of a dead cat reconstructed with an average classical/quantum intensity ratio of 5.5. Scale bar, 400 μm.

While the classical source does not contribute to the intensity-correlation measurement, the presence of uncorrelated photons does reduce the SNR in the measured Γ. Figure 4A shows the decrease of the SNR with the increase of the average intensities ratio between classical and quantum illumination, *I*_cl_/*I*_qu_, together with its theoretical model (see Methods). In this experiment, the camera is illuminated homogeneously with both quantum and classical light (Fig. 4B). SNR values are measured on minus-coordinate projections of Γ that represent the probability of detecting two photons from a pair at two pixels separated by a distance **r**_**1**_ − **r**_**2**_. Figure 4 (C and E) shows minus-coordinate projections of Γ acquired respectively at *I*_cl_/*I*_qu_ = 0 and *I*_cl_/*I*_qu_ = 11. The central peaks are clear signatures of position correlations between pairs of photons (*11*, *12*). As shown in Fig. 4D, this peak disappears when the camera is illuminated only by classical light, i.e., *I*_cl_/*I*_qu_ = +∞. As can be seen, an SNR >1 is maintained over a very wide range of classical illumination intensity levels, even when this is 10× higher than the quantum illumination level, thus indicating that the proposed technique is robust.

## DISCUSSION

In conclusion, we have demonstrated the separation of spatial information carried by quantum light (correlated photons) from that carried by classical light (uncorrelated photons) by intensity correlation measurements. For this, we exploited the existence of spatial correlations between pairs of photons generated by SPDC that are absent in classical coherent light. We also showed that the presence of classical light only decreases the quality of a reconstructed image but does not change its shape. This novel approach may play an important role for quantum imaging in natural environments, where the object and the camera are contaminated by classical noise or spurious photons. Moreover, the ability to mix and distinguish information carried by quantum and classical light may have an important impact in quantum communications (*18*). For example, an image encrypted with correlated photons can be hidden from detectors performing conventional intensity measurements when mixed with a classical image. This work paves the way toward the use of mixed light sources composed of both quantum and classical light for improving imaging (*24*) and communication technologies (*25*).

## METHODS

### Image reconstruction process

The camera was an EMCCD Andor iXon Ultra 897 and was operated at −60°C with a horizontal pixel readout rate of 17 MHz, a vertical pixel shift every 0.3 μs, and a vertical clock amplitude voltage of +4 V above the factory setting. In each acquisition, *N* frames are collected with an exposure time of τ = 6 ms. No threshold was applied, and all calculations were performed directly using gray values returned by the camera (*26*). For **r**_**2**_ ≠ **r**_**1**_, Γ(**r**_**1**_, **r**_**2**_) was calculated using the formula

$$\Gamma (r_{1},r_{2})=\langle I(r_{1})I(r_{2})\rangle -\langle I(r_{1})\rangle \langle I(r_{2})\rangle$$ (2)

The first term is the average intensity product

$$\langle I(r_{1})I(r_{2})\rangle =\underset{N\to +\infty }{\text{lim}}\frac{1}{N}\sum_{l=1}^{N}I_{l}(r_{1})I_{l}(r_{2})$$ (3)

where *I_l_*(**r**_**1**_) [*I_l_*(**r**_**2**_)] corresponds to the intensity value measured at pixel **r**_**1**_ [**r**_**2**_] in the *j*th frame. Experimentally, this term is estimated by multiplying intensity values in each frame and averaging over a large number of frames (typically *N* on the order of 10^6^ to 10^7^). Intensity correlations in this term originate from detections of both real coincidence (two photons from the same entangled pair) and accidental coincidence (two photons from different entangled pairs). The second term in Eq. 2 is defined as

$$\langle I(r_{1})\rangle \langle I(r_{2})\rangle =\underset{N\to +\infty }{\text{lim}}\frac{1}{N^{2}}\sum_{l=1}^{N}\sum_{l\prime =1}^{N}I_{l}(r_{1})I_{l\prime }(r_{2})$$ (4)

Experimentally, this term is estimated by multiplying intensity values between successive frames and averaging over a large number of frames

$$\langle I(r_{1})\rangle \langle I(r_{2})\rangle \approx \frac{1}{N}\sum_{l=1}^{N}I_{l}(r_{1})I_{l+1}(r_{2})$$ (5)

Since there is zero probability for two photons from the same entangled pair to be detected in two different images, intensity correlations in this term originate only from photons from different entangled pairs (accidental coincidence). A subtraction between these two terms (Eq. 2) leaves only genuine coincidences, which is proportional to the joint probability distribution of photon pairs. Moreover, the use of intensity products between successive frames, rather than the products of the averaged intensities, allows the reduction of artifacts such as spatial distortions in the retrieved Γ that are due to fluctuations of the camera amplification gain during the time of an acquisition (*26*).

Since Eq. 2 is only valid for **r**_**2**_ ≠ **r**_**1**_, diagonal values Γ(**r**, **r**) are approximated to intensity correlation values between neighboring pixels Γ(**r**, **r**) ≈ Γ(**r**, **r** + δ**r**), where δ**r** = −δ **e**_**x**_ with δ = 16 μm and **e**_**x**_ is a unit vector. This approximation is justified because the Andor Ultra 897 has a fill factor near 100%, and the correlation width on the camera is estimated to be σ*_r_* ≈ 10 μm (*27*). More details about the image reconstruction process are provided in sections S1 and S2.

**Fig. 5:**
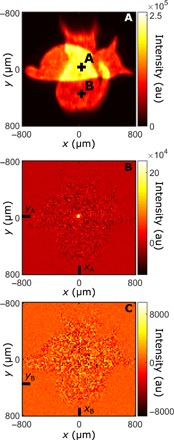
Conditional projections. Direct-intensity image (**A**) measured under simultaneous illumination of classical and quantum light. Conditional image Γ(**r**∣**A**) (**B**) shows an intense peak centered around position **A**. Conditional images Γ(**r**∣**B**) (**C**) are null and flat.

A convenient method to visualize Γ is to use conditional projections. The conditional projection relative to an arbitrarily chosen position **A**, denoted Γ(**r**∣**A**), is an image of intensity correlations between any position **r** and the position **A**. For example, two positions **A** and **B** are selected in the direct-intensity image in Fig. 5A, and their corresponding conditional projections are shown in Fig. 5 (B and C). Γ(**r**∣**A**) shows an intense peak demonstrating that photon pairs from the SPDC source are transmitted together through the object around position **A**. On the contrary, the flat and null pattern of Γ(**r**∣**B**) shows that both photons are absorbed by the object around position **B**.

### Signal-to-noise ratio

We define the SNR as the ratio between the central peak intensity and the variance of the noise surrounding it in the minus-coordinates projection of Γ. This projection is defined as

$$P^{\Gamma }(r^{-})=\int \Gamma (r,r+r^{-})dr$$ (6)

The SNR formula is derived by adapting the approach described in (*28*)

$$\text{SNR}=\alpha \frac{\sqrt{N}\eta }{2}{\left[1+\frac{\sigma_{0}^{2}+I_{\text{cl}}}{\beta (I_{\text{qu}}-\mu_{0})}\right]}^{-1}$$ (7)

where *N* is the number of images acquired, η is the quantum efficiency of the camera sensor, and μ_0_ and σ_0_ are the camera electronic noise mean value and SD, respectively. α and β are two parameters that depend on the shape of Γ and on the amplification process performed by the camera, respectively. *I*_qu_ and *I*_cl_ are intensity values of quantum and classical illuminations, respectively, averaged over all camera pixels, in gray-level units (gl) (16-bit encoding). In Fig. 4, experiments are performed with *N* = 251,600 and *I*_qu_ = 939 gl. Electrical noise parameters μ_0_ = 167 gl and σ_0_ = 32 gl are estimated independently and η ≈ 0.7 is provided by Andor. Last, fitting experimental data with the theoretical model (blue dashed curve in Fig. 4A) returns parameters α = 3.02 ± 0.22 and β = 0.93 ± 0.20 with *R*^2^ = 0.9955.

## Supplementary Material

http://advances.sciencemag.org/cgi/content/full/5/10/eaax0307/DC1

Download PDF

Quantum image distillation

## SUPPLEMENTARY MATERIALS

Supplementary material for this article is available at http://advances.sciencemag.org/cgi/content/full/5/10/eaax0307/DC1 Section S1. Theory Section S2. Measurement of Γ (**r**, **r**) Section S3. Projections of Γ
