# Supplementary material for: Quantum image distillation
Source: Sci Adv. 2019 Oct 18;5(10):eaax0307. doi: 10.1126/sciadv.aax0307 (PMC6799981; doi:10.1126/sciadv.aax0307)
Supplement: http://advances.sciencemag.org/cgi/content/full/5/10/eaax0307/DC1 [file supp_5_10_eaax0307__index.html]

Science Advances | Science AdvancesAAASSearchScience AdvancesMenu

## Supplementary Materials

**This PDF file includes:**

- Section S1. Theory
- Section S2. Measurement of Γ (**r**, **r**)
- Section S3. Projections of Γ

Download PDF

**Files in this Data Supplement:**

- Adobe PDF - aax0307\_SM.pdf
