# Supplementary material for: Quantum image distillation
Source: Sci Adv. 2019 Oct 18;5(10):eaax0307. doi: 10.1126/sciadv.aax0307 (PMC6799981; doi:10.1126/sciadv.aax0307)
Supplement: Download PDF [file aax0307_SM.pdf]

Science Advances
AAAS

advances.sciencemag.org/cgi/content/full/5/10/eaax0307/DC1

Supplementary Materials for

Quantum image distillation

Hugo Defienne*, Matthew Reichert, Jason W. Fleischer, Daniele Faccio*

*Corresponding author. Email: hugo.defienne@glasgow.ac.uk (H.D.); daniele.faccio@glasgow.ac.uk (D.F.)

Published 18 October 2019, *Sci. Adv.* **5**, eaax0307 (2019)
DOI: 10.1126/sciadv.aax0307

This PDF file includes:

Section S1. Theory
Section S2. Measurement of Γ (**r**, **r**)
Section S3. Projections of Γ

SECTION S1. THEORY

This section provides a brief overview of the theory that underlies our image processing technique. A complete description can be found in [27].

We consider the case of a camera illuminated by a source of spatially-entangled photon-pairs, similar to the one shown in Figure 1. Photon-pairs are described by a two-photon wavefunction $\phi(\mathbf{r_1}, \mathbf{r_2})$, where $\mathbf{r_1}$ and $\mathbf{r_2}$ are camera pixels positions. At each camera pixel, photons are converted into intensity values in two steps:

1. Photons are transformed into photo-electrons by a photo-sensitive screen of quantum efficiency η

2. Photo-electrons are transformed into intensity values I_k by an amplification register. For k photo-electrons at the input of the register, the camera returns an average grey value that is proportional to k: $I_k = Ak + x_0$, where x_0 is an electronic noise mean value and A is an amplification parameter.

The camera acquires a set of N images $\{I_l\}_{l \in [\![1,N]\!]}$ using a fixed exposure time. $\langle I(\mathbf{r}) \rangle$ is defined as the mean intensity value measured at pixel $\mathbf{r}$:

$$\langle I(\mathbf{r}) \rangle = \lim_{N \to \infty} \frac{1}{N} \sum_{l=1}^{N} I_l(\mathbf{r}) \tag{1}$$

$\langle I(\mathbf{r_1}) I(\mathbf{r_2}) \rangle$ is defined as the mean intensity product value measured between pixels $\mathbf{r_1}$ and $\mathbf{r_2}$:

$$\langle I(\mathbf{r_1}) I(\mathbf{r_2}) \rangle = \lim_{N \to \infty} \frac{1}{N} \sum_{l=1}^{N} I_l(\mathbf{r_1}) I_l(\mathbf{r_2}) \tag{2}$$

The theoretical analysis is performed under the following assumptions:

i. Pump laser is operating above threshold to ensure a Poisson distribution of pump photons

ii. Pump laser power is low enough to ensure that > 2 photons generation process in the crystal are negligible

iii. Coherence time of photon-pairs is much smaller than the time between two successive images

iv. Cross-talk between pixels is negligible

Following the reasoning detailed in the Appendix E of the supplementary document of [27], $\langle I(\mathbf{r}) \rangle$ and $\langle I(\mathbf{r_1}) I(\mathbf{r_2}) \rangle$ are written in function of the camera parameters (x_0 and A) and the joint probability distribution of photon-pairs $|\phi(\mathbf{r_1}, \mathbf{r_2})|^2$. On the one hand, $\langle I(\mathbf{r}) \rangle$ is written as

$$\langle I(\mathbf{r}) \rangle = x_0 + 2A\bar{m}\eta P_m(\mathbf{r}) \tag{3}$$

where $\bar{m}$ is the mean photon-pair rate and $P_m(\mathbf{r}) = \int |\phi(\mathbf{r}, \mathbf{r'})|^2 d\mathbf{r'}$ is the probability of detecting a photon at pixel $\mathbf{r}$ (i.e. marginal probability). On the other hand, for $\mathbf{r_1} \neq \mathbf{r_2}$, $\langle I(\mathbf{r_1}) I(\mathbf{r_2}) \rangle$ is written as

$$\begin{aligned} \langle I(\mathbf{r_1}) I(\mathbf{r_2}) \rangle = x_0^2 \\ +2Ax_0\bar{m}\eta[P_m(\mathbf{r_1}) + P_m(\mathbf{r_2})] \\ +4A^2\bar{m}^2\eta^2 P_m(\mathbf{r_1}) P_m(\mathbf{r_2}) \\ +4A^2\bar{m}\eta^2 |\phi(\mathbf{r_1}, \mathbf{r_2})|^2 \end{aligned} \tag{4}$$

Finally, the joint probability distribution $|\phi(\mathbf{r_1}, \mathbf{r_2})|^2$ can be written as

$$|\phi(\mathbf{r_1}, \mathbf{r_2})|^2 = \frac{\langle I(\mathbf{r_1}) I(\mathbf{r_2}) \rangle - \langle I(\mathbf{r_1}) \rangle \langle I(\mathbf{r_2}) \rangle}{4A^2\bar{m}\eta^2} \tag{5}$$

While it is commonly thought that photon counting is necessary to compute the joint probability distribution, this result shows that simple operation of a camera without threshold also enables its measurement.
However, this result is only valid for $\mathbf{r_1} \neq \mathbf{r_2}$. As described in Appendix H of the supplementary document of [27], $\langle I(\mathbf{r})^2 \rangle$ can be written as:

$$\begin{aligned} \langle I(\mathbf{r})^2 \rangle = 2A^2\bar{m}\eta^2 \Gamma(\mathbf{r}, \mathbf{r}) + 4A^2\bar{m}\eta^2 P_m(\mathbf{r})^2 \\ + 4(A^2 + Ax_0)\bar{m}\eta P_m(\mathbf{r}) + \sigma_0 + x_0^2 \end{aligned} \tag{6}$$

where σ_0 is the standard deviation of the camera electronic noise. As a result, $\langle I(\mathbf{r})^2 \rangle \neq \langle I(\mathbf{r}) \rangle^2$ and equation (4) is not valid for $\mathbf{r_1} = \mathbf{r_2}$. In our experiment, $\Gamma(\mathbf{r}, \mathbf{r})$ is estimated using the approximation $\Gamma(\mathbf{r}, \mathbf{r}) = \Gamma(\mathbf{r}, \mathbf{r} + \delta\mathbf{r})$, where $\delta\mathbf{r} = -\delta\, \mathbf{e_x}$ with $\delta = 16\mu m$ (pixel width) and $\mathbf{e_x}$ is an unit vector.

SECTION S2. MEASUREMENT OF $\Gamma(\mathbf{r}, \mathbf{r})$

In our experiment, the camera is an EMCCD Andor Ixon Ultra 897. It was operated at −60°C, with a horizontal pixel shift readout rate of 17Mhz, a vertical pixel shift every 0.3 μs and a vertical clock amplitude voltage of +4V above the factory setting. Exposure time

is set to 6ms. All assumptions enumerated in section I are verified: pump laser operates above threshold with a power of $\sim 50mW$ [(i) and (ii)], coherent time of the pairs ($\sim 1ps$) is much smaller than the time between two successive frames ($\sim 4ms$) (iii) and cross-talk between pixels is negligible (iv). In the following, we describe step-by-step the technique to measure $\Gamma(\mathbf{r},\mathbf{r})$:

1. Acquisition of a set of N images $\{I_l\}_{l\in[\![1,N]\!]}$ at fixed exposure time τ = 6ms, with N on the order of 10^{6-7}.

2. Estimation of the first term of equation (2) by multiplying pixel values in each image by themselves and averaging over the set:

$$\langle I(\mathbf{r_1})I(\mathbf{r_2})\rangle \approx \frac{1}{N}\sum_{l=1}^{N} I_l(\mathbf{r_1})I_l(\mathbf{r_2}) \tag{7}$$

in which $\mathbf{r_1}$ and $\mathbf{r_2}$ are pixel positions [$\mathbf{r_1} \neq \mathbf{r_2}$].

3. Estimation of the second term of equation (2) by multiplying pixel values in the lth image by those of the following image $l+1^{th}$ and average over the set:

$$\langle I(\mathbf{r_1})\rangle\langle I(\mathbf{r_2})\rangle \approx \frac{1}{N^2}\sum_{l=1}^{N} I_l(\mathbf{r_1})I_{l+1}(\mathbf{r_2})$$

By definition, $\langle I(\mathbf{r_1})\rangle\langle I(\mathbf{r_2})\rangle$ equals the limit $N \to +\infty$ for the following summation:

$$\frac{1}{N^2}\sum_{l=1}^{N}\sum_{l'=1}^{N} I_l(\mathbf{r_1})I_{l'}(\mathbf{r_2}) = \frac{1}{N^2}\sum_{l=1}^{N} I_l(\mathbf{r_1})I_l(\mathbf{r_2}) + \frac{1}{N^2}\sum_{l\neq l'}^{N} I_l(\mathbf{r_1})I_{l'}(\mathbf{r_2}) \tag{8}$$

The first term in equation 8 can be written as:

$$\frac{1}{N}[\frac{1}{N}\sum_{l=1}^{N} I_l(\mathbf{r_1})I_l(\mathbf{r_2})] = o\left(\frac{1}{N}\right) \tag{9}$$

because by definition of $\langle I(\mathbf{r_1})I(\mathbf{r_2})\rangle$, the serie $\frac{1}{N}\sum_{l=1}^{N} I_l(\mathbf{r_1})I_l(\mathbf{r_2})$. The second term in equation 8 is an estimation of the mean value of intensity product between different frames $\langle I_l(\mathbf{r_1})I_{l\neq l'}(\mathbf{r_2})\rangle$. Because the probability for two photons of the same pair to be detected in two different frames is null (coherent time much smaller than camera readout time), intensity values in different frames are independent with each other. In consequence, $\langle I_l(\mathbf{r_1})I_{l\neq l'}(\mathbf{r_2})\rangle$ can be estimated using only successive frames by calculating the sum $\frac{1}{N^2}\sum_{l=1}^{N} I_l(\mathbf{r_1})I_{l+1}(\mathbf{r_2})$. Experimentally, the use of successive frames to estimate $\langle I(\mathbf{r_1})\rangle\langle I(\mathbf{r_2})\rangle$ rather than the complete set has the advantage of reducing artifacts as spatial distortions in the measured Γ, mainly due to fluctuations of the amplification gain of the camera [27].

4. Subtraction between these two terms:

$$\Gamma(\mathbf{r_1},\mathbf{r_2}) \approx \frac{1}{N}\sum_{l=1}^{N} I_l(\mathbf{r_1})I_l(\mathbf{r_2}) - \frac{1}{N^2}\sum_{l\neq l'}^{N} I_l(\mathbf{r_1})I_{l'}(\mathbf{r_2}) \tag{10}$$

5. As shown in Section I, equation 5 is only valid for $\mathbf{r_1} \neq \mathbf{r_2}$. Estimation of the intensity correlation values $\Gamma(\mathbf{r},\mathbf{r})$ from those measured between pixel $\mathbf{r} = (x,y)$ is then performed using neighbouring pixels $\mathbf{r}' = (x-\delta,y)$ [$\delta = 16\mu m$ = pixel size]:

$$\Gamma(\mathbf{r},\mathbf{r}) \approx \Gamma((x,y),(x-\delta,y)) \tag{11}$$

In our experiment, this approximation is valid because the fill factor of the Andor Ixon Ultra is near 100% and the position correlation width on the camera is estimated from the thickness of the crystal to be $\sigma_r \approx 10\mu m$ [28].

SECTION S3. PROJECTIONS OF Γ

$\Gamma(\mathbf{r_1},\mathbf{r_2}) = \Gamma((x_1,y_1)\ (x_2,y_2))$ is a 4-dimensional matrix. Its information content can be visualized using two types of projections:

1. Conditional projection relative to an arbitrarily chosen position $\mathbf{r}'$, defined as

$$\Gamma(\mathbf{r}|\mathbf{r}') = \frac{\Gamma(\mathbf{r},\mathbf{r}')}{\sum_{\mathbf{r}'}\Gamma(\mathbf{r},\mathbf{r}')} \tag{12}$$

It represents the probability of detecting a photon from a pair at position $\mathbf{r}$ under the condition that another photon is detected at $\mathbf{r}'$.

2. The minus-coordinate projection, defined as

$$P_-^{\Gamma}(\mathbf{r}_-) = \sum_{\mathbf{r}}\Gamma(\mathbf{r}_- + \mathbf{r},\mathbf{r}) \tag{13}$$

It represents the probability for two photons of a pair to be detected in coincidence between pairs of pixels separated by an oriented distance $\mathbf{r}_-$.
